# Micronutrients Involved in One-Carbon Metabolism and Risk of Breast Cancer Subtypes

**DOI:** 10.1371/journal.pone.0138318

**Published:** 2015-09-16

**Authors:** Ilaria Cancarini, Vittorio Krogh, Claudia Agnoli, Sara Grioni, Giuseppe Matullo, Valeria Pala, Samuele Pedraglio, Paolo Contiero, Cristina Riva, Paola Muti, Sabina Sieri

**Affiliations:** 1 Department of Preventive and Predictive Medicine, Epidemiology and Prevention Unit, Fondazione IRCSS Istituto Nazionale dei Tumori, Milan, Italy; 2 Department of Medical Sciences, University of Torino and Human Genetics Foundation (HuGeF), Turin, Italy; 3 Department of Preventive and Predictive Medicine, Environmental Epidemiology Unit, Fondazione IRCSS Istituto Nazionale dei Tumori, Milan, Italy; 4 Department of Surgical and Morphological Sciences, University of Insubria, Varese, Italy; 5 Department of Oncology, Faculty of Health Science, McMaster University, Hamilton, Ontario, Canada; Tufts University, UNITED STATES

## Abstract

**Background:**

Vitamins involved in one-carbon metabolism are hypothesized to influence breast cancer (BC) risk. However, epidemiologic studies that examined associations between B vitamin intake and BC risk have provided inconsistent results. We prospectively examined, in the Italian ORDET cohort, whether B vitamin consumption was associated with risk of BC and BC subtypes.

**Methods:**

After a mean follow-up of 16.5 years, 391 BCs were diagnosed among 10,786 cohort women. B vitamin intakes were estimated from food frequency questionnaires. Cox proportional hazard models adjusted for energy intake and confounders, estimated hazard ratios (HR) with 95% confidence intervals (CIs) for BC according to intake.

**Results:**

RRs were 0.61 (95% CI 0.38–0.97 highest vs. lowest quartile; P trend 0.025) for thiamine; 0.48 (95% CI 0.32–0.71; P trend <0.001) for riboflavin; 0.59 (95% CI 0.39–0.90; P trend 0.008) for vitamin B6, and 0.65 (95% CI 0.44–0.95; P trend 0.021) for folate. As regards risk of BC subtypes, high riboflavin and folate were significantly associated with lower risk of estrogen receptor positive (ER+) and progesterone receptor positive (PR+) cancers, and high thiamine was associated with lower risk of ER-PR- cancers. High riboflavin was associated with lower risk of both HER2+ and HER2- cancers, high folate with lower risk of HER2- disease, and high thiamine with HER2+ disease.

**Conclusions:**

These findings support protective effects of thiamine and one-carbon metabolism vitamins (folate, riboflavin, and vitamin B6) against BC in general; while folate may also protect against ER+PR+ and HER2- disease; and thiamine against ER-PR-, and HER2+ disease.

## Introduction

One-carbon metabolism is a network of biochemical pathways that provides methyl groups for a variety of essential biomolecules and biological processes. Disruption of one-carbon metabolism can interfere with DNA replication, DNA repair, and regulation of gene expression through methylation, each of which could promote carcinogenesis. The B vitamin folate/folic acid is a major dietary source of methyl groups, whereas riboflavin (vitamin B2) and vitamin B6 are essential cofactors for enzymes involved in one-carbon metabolism that may influence methyl group bioavailability. Thus it is reasonable that dietary intake of B vitamins may influence the development and progression of cancer, and dietary changes in the intake of these nutrients may modify the risk of cancer [1–4].

While many epidemiological studies have investigated the effect of dietary folate intake on breast cancer risk, relatively few studies have evaluated the influence of intake of other B vitamins on risk. Finding on folate have been inconsistent [5]. Two 2014 meta-analyses of observational studies[6,7] found that evidence from prospective epidemiological studies was inconclusive, while data from case-control studies[7] suggested that high folate had a significant protective effect against breast cancer. Studies that evaluated the influence of intake of other B vitamins on breast cancer risk have also produced mixed results [8–16].

Breast cancer is now classified into subtypes based in theory on gene expression profiles but determined in clinical practice by the expression of estrogen receptor (ER), progesterone receptor (PR), overexpression of human epidermal growth factor receptor 2 (HER2) and the Ki67 proliferation index [17].

These subtypes differ in prognosis and factors that influence their occurrence [18,19]. Only limited data on the influence of intake of micronutrients involved in one-carbon metabolism on the risk of developing breast cancer subtypes are available. Two recent meta-analyses that investigated folate did not find any significant associations [5,6].

The aim of the present study was to investigate the hypothesis that breast cancer subtypes defined by ER, PR and HER2 status (but not Ki67, since we had no data available) have differing etiologic pathways differentially influenced by intake of nutrients involved in one-carbon metabolism. We investigated women enrolled in the Italian ORDET (H**OR**mones and **D**iet in the **E**tiology of Breast **T**umours) study.

## Materials and Methods

### Study population

The ORDET cohort consisted of 10,786 healthy women, residents of Varese Province in northern Italy, recruited voluntarily between June 1987 and June 1992. Age at recruitment was 35–69 years. Women taking hormone therapy in the three months before recruitment, with chronic or acute liver disease, with a history of cancer, or who had undergone bilateral ovariectomy, were not eligible.

The study protocol was approved by the ethics committee of the Fondazione IRCCS Istituto Nazionale dei Tumori (Milan, Italy). The study complies with the Helsinki Declaration, and participants gave written informed consent to use clinical data for research.

At recruitment, information on lifestyle, menstrual history and reproductive history was collected; height, weight, and waist and hip circumferences were measured; and blood and urine samples were collected. The women also completed a self-administered semi-quantitative food frequency questionnaire (FFQ) [20].

The ORDET database was linked to the local Varese Cancer Registry, which is considered a high quality registry with less than 3% of cancers identified only through death certificates [21], to ascertain incident breast cancer cases (invasive and in situ) up to December 2006. Local residential and mortality databases were accessed to check vital status. Information on the ER, PR and HER2 status of cancers was obtained from electronic pathology reports. To standardize the quantification of receptor status, the following criteria for positivity were adopted: ≥10% cells stained, any ‘positive’ description, ≥20 fmol/mg, Allred score ≥3, immunoreactive score ≥2, or H-score ≥10 [22]. HER2 was considered overexpressed (positive) when >30% of cancer cells showed complete intense membrane staining (score 3+ according to ASCO 2007 guidelines) [23].

The FFQ only became available 30 months after starting recruitment. Women recruited at the beginning (n = 1500) did not complete the FFQ and were excluded from the present analysis. We also excluded women found to have a cancer diagnosis before recruitment, or who were lost to early follow-up, leaving 9093 women potentially eligible women. We subsequently excluded women for whom variables used as covariates in the statistical model were missing, and those for whom the ratio of total energy intake (determined from the FFQ) to basal metabolic rate (determined by the Harris-Benedict equation)[24] was in the first or last half-percentiles of the distribution: this to reduce the effect of implausible extreme values on the analysis. We therefore analysed 9009 women including 391 incident breast cancer cases.

### Food frequency questionnaire

Dietary habits over the preceding year were assessed using a validated semi-quantitative FFQ, as described in detail elsewhere [20]. Volunteers completed the FFQ, on their own, at recruitment, with immediate review by a nurse/volunteer to draw attention to any missing items. The FFQ consisted of 107 food items and included photos with two or three sample dishes of definite sizes, or references to standard portion sizes. The composition in nutrients of individual food items was obtained from Italian food composition tables [25] and the average intakes of macro and micronutrients for each volunteer were estimated. Few women in the ORDET cohort used B vitamin supplements at recruitment (0.58%) and while folic acid fortification of grain products is mandatory in the US, this is not the case in Italy. In this study we only estimated vitamin B intake from dietary sources excluding supplements.

### Statistical analyses

Hazard ratios (HRs) with 95% confidence intervals (CIs) of developing breast cancer in relation to intakes of thiamine (B1), riboflavin (B2), niacin (B3), vitamin B6, and folate (B9) were estimated by multivariable Cox proportional hazard modelling with age as primary time variable. Vitamin intakes were categorized into quartiles based on the distribution of intake in the whole cohort, with the lowest quartile as reference. HRs were also calculated for 1 standard deviation increments in vitamin intake as a continuous variable.

We ran a minimally adjusted model, with energy intake as covariate, and a fully-adjusted model with the following additional covariates: height (m), waist-to-hip ratio, age at menarche (years), menopausal status (pre-, peri-, postmenopausal), oral contraception use (yes or no), parity (nulliparous, 1–2 children, >2 children), years of education, family history of breast cancer (yes or no), and alcohol intake (g/day). Tests for linear trend were calculated by assigning an ordinal number to each quartile.

The proportional hazards assumption for B vitamins and all other covariates in relation to breast cancer risk was tested using the Grambsch and Therneau method [26]. In all cases, the proportional hazards assumption was satisfied.

In additional analyses, we tested whether effects of vitamin intake differed with alcohol intake, menopausal status, and BMI. To do this we performed stratified analyses using product terms (1 for alcohol intake <12g/d and 0 for alcohol ≥12 g/d; 1 for pre-menopause and 0 for menopause, 1 for BMI ≤25 and 0 for >25) that were multiplied by vitamin intake as a continuous variable. These models were adjusted with the same covariates as in the fully-adjusted model, with appropriate exclusions (menopausal status excluded in the model stratifying by menopausal status; alcohol intake excluded in the model stratifying by alcohol intake). The significance of interactions was assessed using a likelihood ratio test that compared the model that included the product term and the model that did not include it.

We investigated associations between vitamin intake and breast cancer in general, and also breast cancer subtypes defined by ER status (ER+, ER-), PR status (PR+, PR-), combined ER and PR status (ER+PR+, ER-PR-) and HER2 status (HER2+, HER2-).

The heterogeneity of associations according to receptor status was assessed using the data augmentation method [27] in which the difference in log likelihood between a model with receptor status-specific variables, and a model with a single HR estimate for each receptor status category, was compared to a chi-square distribution with 1 degree of freedom (comparison between receptor positive and receptor negative). In these analyses, women who developed a competing breast cancer subtype or had missing receptor status were censored at time of diagnosis.

## Results

After a mean follow up of 16.3 years, 391 breast cancer cases were identified in the cohort (362 invasive and 29 in situ). Of these, 282 were ER+ and 82 ER- (27 unknown); 236 were PR+ and 126 PR- (29 unknown); and 62 HER2+ and 273 HER2- (56 unknown).

The distributions of vitamin intake and breast cancer risk factors in the cohort at recruitment are shown in **Table 1** according to disease status. Women who developed breast cancer were more educated, used less oral contraceptives, more often had a family history of breast cancer, and overall had a lower intake of B vitamins than those who did not.

**Table 1:** Baseline distribution of nutrients and factors influencing breast cancer risk by disease status in ORDET women.

	Cases (n = 391)	Non-cases (n = 8618)
**Age in years** (SD*)	49.62 (8.18)	48.47 (8.59)
**Weight in** kg (SD*)	63.65 (11.37)	62.97 (10.89)
**Height** in m (SD*)	1.58 (0.06)	1.58 (0.06)
**Waist-hip ratio (**SD*)	0.79 (0.07)	0.79 (0.06)
**Age (years) at menarche** (SD* **)**	12.84 (1.57)	12.85 (1.52)
**Menopausal status** (%)		
Premenopausal	53.71	56.35
Perimenopausal	5.63	5.83
Postmenopausal	39.64	36.80
**Parity** (%)		
Nulliparous	11.25	10.88
1–2 children	66.50	65.99
>2 children	22.25	23.13
**Education** (% >8 years)	30.43	28.22
**Oral contraceptive use** (% yes)	29.92	33.46
**Family history of BC** (% yes)	10.74	7.12
**Thiamine** (mg/day)	0.93 (0.27)	0.96 (0.28)
**Riboflavin** (mg/day)	1.37 (0.50)	1.43 (0.46)
**Niacin** (mg/day)	16.01 (5.00)	16.56 (5.25)
**Vitamin B6** (mg/day)	1.92 (0.59)	2.01 (0.60)
**Folic acid** (μg/day)	294.74 (101.78)	306.64 (101.50)
**Energy (**kcal/day)	1735.30 (503.68)	1779.82 (509.81)
**Alcohol** (g/day)	10.15 (13.76)	10.04 (13.20)

* SD = standard deviation

Multivariable-adjusted HRs for developing breast cancer by quartiles of B vitamin intake are shown in **Table 2**. Women in highest thiamine intake quartile had a significantly lower risk of breast cancer than those in the lowest quartile (RR 0.61; 95%CI 0.38–0.97; P trend 0.025; fully-adjusted model). A similar significant effect was seen for riboflavin (HR 0.48; 95% CI 0.32–0.71; P trend <0.001, fully-adjusted model), which was also present when riboflavin intake was considered as a continuous variable (HR 0.80; 95% CI 0.67–0.94; fully-adjusted model). A similar significant effect was also seen for vitamin B6 (HR 0.59; 95% CI 0.39–0.90; P trend 0.008; fully-adjusted model), which again was present when vitamin B6 was considered as a continuous variable (HR 0.77; 95% CI 0.65–0.92; fully-adjusted model). Finally, the highest quartile of folate intake was also associated with lower breast cancer risk than the lowest quartile (HR 0.65; 95% CI 0.44–0.95; P trend 0.021; fully-adjusted model). No significant association of niacin intake with breast cancer was found.

**Table 2:** HRs (95% CIs) of breast cancer in relation to quartiles of B vitamin intake in ORDET women.

	Quartiles					
	I	II	III	IV	P trend#	Continuous##
**Thiamine**						
Median intake (mg/day)	0.67	0.86	1.02	1.27		
Cases/person-year	122/34504	96/34507	87/34326	86/34662		
Crude HR (95% CI)*	1	0.77 (0.58–1.03)	0.68 (0.49–0.96)	0.64 (0.40–1.01)	0.036	0.89 (0.72–1.10)
Multivariable HR (95% CI)**	1	0.75 (0.56–1.01)	0.66 (0.47–0.94)	0.61 (0.38–0.97)	0.025	0.87 (0.69–1.09)
**Riboflavin**						
Median intake (mg/day)	0.96	1.24	1.51	1.95		
Cases/person-year	140/34834	87/34317	80/34357	84/34491		
Crude HR (95% CI)	1	0.60 (0.45–0.79)	0.53 (0.38–0.72)	0.50 (0.34–0.74)	<0.001	0.81 (0.68–0.95)
Multivariable HR (95% CI)	1	0.57 (0.43–0.76)	0.50 (0.37–0.69)	0.48 (0.32–0.71)	<0.001	0.80 (0.67–0.94)
**Niacin**						
Median intake (mg/day)	11.02	14.46	17.63	22.17		
Cases/person-year	105/34232	106/34606	92/34514	88/34648		
Crude HR (95% CI)	1	1.03 (0.78–1.37)	0.92 (0.67–1.27)	0.92 (0.62–1.36)	0.556	0.88 (0.74–1.03)
Multivariable HR (95% CI)	1	1.01 (0.76–1.35)	0.91 (0.66–1.26)	0.91 (0.61–1.36)	0.546	0.88 (0.74–1.04)
**Vitamin B6**						
Median intake (mg/day)	1.37	1.78	2.12	2.66		
Cases/person-year	119/34463	101/34405	89/34528	82/34604		391/8618
Crude HR (95% CI)	1	0.82 (0.62–1.09)	0.70 (0.51–0.96)	0.61 (0.41–0.92)	0.011	0.78 (0.66–0.93)
Multivariable HR (95% CI)	1	0.81 (0.61–1.06)	0.68 (0.49–0.94)	0.59 (0.39–0.90)	0.008	0.77 (0.65–0.92)
**Folate**						
Median intake (μg/day)	198.55	265.44	324.56	420.56		
Cases/person-year	112/34419	108/34593	95/34625	76/34362		
Crude HR (95% CI)	1	0.95 (0.72–1.26)	0.82 (0.60–1.12)	0.65 (0.45–0.95)	0.021	0.87 (0.76–1.01)
Multivariable HR (95% CI)	1	0.94 (0.71–1.24)	0.81 (0.59–1.10)	0.65 (0.44–0.95)	0.021	0.87 (0.75–1.03)

* Adjusted by energy intake.

** Adjusted by height, waist hip ratio, age at menarche, menopausal status, oral contraceptive use, parity, education, family history of breast cancer, energy intake and alcohol intake.

# Tests for linear trend were calculated by assigning an ordinal number to each quartile.

## HR of developing breast cancer per 1 SD increase in vitamin intake

**Table 3** shows associations between B vitamin intake and breast cancer risk according to ER status. High riboflavin intake was associated with significantly reduced risk of ER+ disease, evident both in the quartile (HR 0.40; 95% CI 0.25–0.65 highest vs. lowest; P trend <0.001) and continuous (HR 0.77; 95% CI 0.63–0.94) models.

**Table 3:** HRs* (95% CIs) of breast cancer by ER and PR status in relation to quartiles of B vitamin intake in ORDET women.

	ER+		ER-		PR+		PR-	
	Cases/person-year	Multivariable HR (95% CI)*	Cases/person-year	Multivariable HR (95% CI)	Cases/person-year	Multivariable HR (95% CI)	Cases/person-year	Multivariable HR (95% CI)
**Thiamine**								
I	90/34246	1	25/33593	1	71/34039	1	43/33788	1
II	72/34283	0.80 (0.57–1.12)	17/33777	0.56 (0.29–1.08)	54/34085	0.76 (0.51–1.11)	35/33973	0.72 (0.44–1.18)
III	61/34083	0.68 (0.45–1.03)	16/33632	0.46 (0.22–0.98)	53/33995	0.74 (0.47–1.16)	25/33732	0.48 (0.26–0.89)
IV	59/34487	0.65 (0.37–1.13)	24/34057	0.51 (0.20–1.33)	58/34426	0.80 (0.44–1.44)	23/34102	0.37 (0.16–0.88)
P trend**		0.087		0.118		0.388		0.012
Continuous#		0.85 (0.65–1.11)		0.88 (0.55–1.41)		0.89 (0.67–1.18)		0.75 (0.50–1.13)
P for heterogeneity##		0.870				0.516		
**Riboflavin**								
I	105/34534	1	28/33742	1	87/34318	1	46/33959	1
II	66/34140	0.57 (0.41–0.80)	13/33629	0.41 (0.21–0.80)	51/33988	0.52 (0.36–0.74)	26/33770	0.55 (0.33–0.91)
III	58/34184	0.49 (0.34–0.71)	17/33811	0.47 (0.24–0.92)	49/34063	0.45 (0.30–0.68)	27/33938	0.58 (0.33–1.01)
IV	53/34241	0.40 (0.25–0.65)	24/33876	0.53 (0.24–1.19)	49/34177	0.38 (0.23–0.63)	27/33928	0.57 (0.29–1.14)
P trend		<0.001		0.129		<0.001		0.103
Continuous#		0.77 (0.63–0.94)		0.89 (0.63–1.25)		0.72 (0.58–0.89)		0.94 (0.71–1.25)
P for heterogeneity##		0.472				0.130		
**Niacin**								
I	75/33964	1	24/33457	1	56/33752	1	42/33658	1
II	80/34387	1.10 (0.79–1.54)	20/33812	0.74 (0.40–1.38)	69/34261	1.23 (0.85–1.79)	32/33949	0.76 (0.47–1.23)
III	70/34318	1.04 (0.71–1.52)	14/33778	0.46 (0.22–0.95)	57/34177	1.06 (0.69–1.62)	27/33918	0.64 (0.37–1.13)
IV	57/34429	0.93 (0.57–1.50)	24/34012	0.64 (0.28–1.44)	54/34356	1.04 (0.62–1.75)	25/34070	0.60 (0.30–1.22)
P trend		0.761		0.155		0.958		0.121
Continuous#		0.89 (0.73–1.08)		0.80 (0.56–1.13)		0.92 (0.75–1.14)		0.75 (0.55–1.02)
P for heterogeneity##		0.601				0.262		
**Vitamin B6**								
I	88/34211	1	25/33575	1	67/33988	1	44/33781	1
II	76/34191	0.84 (0.60–1.16)	19/33633	0.65 (0.35–1.21)	62/34030	0.88 (0.61–1.27)	34/33806	0.72 (0.45–1.16)
III	61/34270	0.67 (0.45–0.98)	15/33829	0.45 (0.22–0.92)	54/34179	0.74 (0.49–1.12)	23/33925	0.47 (0.26–0.85)
IV	57/34427	0.62 (0.38–1.01)	23/34022	0.52 (0.23–1.20)	53/34349	0.70 (0.41–1.18)	25/34084	0.48 (0.23–0.98)
P trend		0.030		0.073		0.133		0.016
Continuous#		0.82 (0.66–1.00)		0.63 (0.44–0.91)		0.88 (0.71–1.10)		0.58 (0.42–0.80)
P for heterogeneity##		0.240				0.031		
**Folate**								
I	83/34188	1	23/33557	1	64/33982	1	41/33752	1
II	82/34353	0.98 (0.71–1.35)	17/33745	0.66 (0.35–1.27)	66/34175	0.98 (0.68–1.41)	32/33917	0.80 (0.49–1.30)
III	69/34415	0.81 (0.57–1.17)	19/33941	0.68 (0.34–1.33)	63/34326	0.90 (0.60–1.33)	26/34042	0.66 (0.38–1.14)
IV	48/34144	0.58 (0.36–0.91)	23/33816	0.73 (0.34–1.60)	43/34063	0.58 (0.35–0.96)	27/33884	0.74 (0.39–1.41)
P trend		0.018		0.467		0.049		0.254
Continuous#		0.84 (0.70–1.00)		0.98 (0.72–1.32)		0.84 (0.69–1.01)		0.93 (0.71–1.20)
P for heterogeneity##		0.378				0.534		

* Adjusted for height, waist-hip-ratio, age at menarche, menopausal status, oral contraceptive use, parity, education, family history of breast cancer, energy intake, and alcohol intake.

** Tests for linear trend calculated by assigning an ordinal number to each quartile.

# HR of developing breast cancer per 1 SD increase in vitamin intake.

## Receptor positive vs. receptor negative (ER+ vs. ER-; PR+ vs. PR-).

High vitamin B6 intake was also associated with reduced risk of ER+ disease: with a significant P trend, and significant by the continuous model (HR 0.82; 95% CI 0.66–1.00).

High folate intake was also associated with a significantly reduced risk of ER+ disease, evident in both the quartile (HR 0.58; 95% CI 0.36–0.91 highest vs. lowest; P trend 0.018) and continuous (HR 0.84; 95% CI 0.70–1.00) models.

No significant associations of thiamine or niacin with ER+ breast cancer were found; neither were significant associations of riboflavin, folate, thiamine or niacin with ER- breast cancer found, although HR estimates were in the same direction as those for ER+ breast cancer. Vitamin B6 intake was significantly associated with reduced risk of ER- disease in the continuous model only (HR 0.63; 95% CI 0.44–0.91).

As regards PR+ disease, high intakes of riboflavin (HR 0.38; 95% CI 0.23–0.63 highest vs. lowest quartile; P trend <0.001) and of folate (HR 0.58; 95% CI 0.35–0.96 highest vs. lowest quartile; P trend, 0.049) were significantly associated with reduced incidence of this cancer subtype in the quartile models; riboflavin also had a significant effect in the continuous model (HR 0.72; 95% CI 0.58–0.89).

As regards PR- disease, high thiamine (HR 0.37; 95% CI 0.16–0.88 highest vs. lowest quartile; P trend 0.012) and high vitamin B6 (HR 0.48; 95% CI 0.23–0.98 highest vs. lowest quartile; P trend 0.016) were significantly associated with lowered risk of this cancer subtype in the quartile models; vitamin B6 was also effective in the continuous model (HR 0.58; 95% CI 0.42–0.80). The test for heterogeneity between PR+ and PR- for vitamin B6 was significant (P = 0.030). No association of riboflavin, niacin, or folate with PR- breast cancer was found.

**Table 4** shows associations between vitamin intake with risk of breast cancer subtypes defined by joint ER and PR status. High intakes of riboflavin and folate were significantly associated with lowered risk of ER+PR+ disease, both in the quartile (HR 0.36; 95% CI 0.21–0.62 highest vs. lowest; P trend <0.001; HR 0.52, 95% CI 0.31–0.90 highest vs. lowest; P trend 0.024, respectively) and the continuous (HR 0.69; 95% CI 0.55–0.88; HR 0.79; 95% CI 0.64–0.97, respectively) models.

**Table 4:** HRs (95% CIs) of breast cancer by ER plus PR status in relation to quartiles of B vitamin intake in ORDET women.

	ER+PR+		ER-PR-	
	Cases/person-year	Multivariable HR (95% CI)*	Cases/person-year	Multivariable HR (95% CI)
**Thiamine**				
I	68/34027	1	22/33581	1
II	50/34055	0.73 (0.49–1.09)	14/33752	0.50 (0.25–1.03)
III	49/33966	0.72 (0.45–1.14)	13/33615	0.40 (0.17–0.91)
IV	50/34400	0.71 (0.38–1.33)	14/34015	0.29 (0.09–0.91)
P trend**		0.254		0.024
Continuous #		0.84 (0.62–1.14)		0.64 (0.37–1.12)
P for Heterogeneity##		0.405		
**Riboflavin**				
I	83/34295	1	24/33720	1
II	47/33962	0.51 (0.35–0.74)	9/33611	0.36 (0.16–0.79)
III	44/34037	0.43 (0.28–0.66)	13/33790	0.47 (0.22–1.02)
IV	43/34154	0.36 (0.21–0.62)	17/33841	0.55 (0.22–1.38)
P trend**		<0.001		0.201
Continuous#		0.69 (0.55–0.88)		0.85 (0.57–1.28)
P for Heterogeneity##		0.379		
**Niacin**				
I	53/33736	1	21/33442	1
II	65/34231	1.24 (0.85–1.83)	17/33793	0.73 (0.37–1.43)
III	51/34148	1.03 (0.66–1.61)	9/33754	0.35 (0.15–0.84)
IV	48/34334	1.03 (0.60–1.78)	16/33974	0.53 (0.20–1.36)
P trend**		0.901		0.072
Continuous#		0.92 (0.74–1.14)		0.71 (0.46–1.07)
P for Heterogeneity##		0.275		
**Vitamin B6**				
I	63/33966	1	21/33554	1
II	58/33999	0.89 (0.61–1.30)	16/33613	0.67 (0.34–1.33)
III	49/34159	0.74 (0.48–1.14)	11/33813	0.41 (0.18–0.94)
IV	47/34325	0.69 (0.40–1.20)	15/33982	0.44 (0.17–1.18)
P trend**		0.136		0.054
Continuous#		0.88 (0.70–1.11)		0.53 (0.34–0.82)
P for Heterogeneity##		0.040		
**Folate**				
I	62/33975	1	21/33550	1
II	61/34148	0.95 (0.65–1.38)	12/33718	0.54 (0.26–1.13)
III	57/34285	0.85 (0.56–1.27)	14/33912	0.59 (0.28–1.26)
IV	37/34040	0.52 (0.31–0.90)	16/33780	0.64 (0.26–1.54)
P trend**		0.024		0.334
Continuous#		0.79 (0.64–0.97)		0.84 (0.58–1.22)
P for Heterogeneity##		0.760		

* Adjusted for height, waist-hip-ratio, age at menarche, menopausal status, oral contraceptive use, parity, education, family history of breast cancer, energy intake, and alcohol intake.

** Tests for linear trend were calculated by assigning an ordinal number to each quartile.

# HR of developing breast cancer per 1 SD increase in vitamin intake.

## ER+PR+ vs. ER-PR-

As regards ER-PR- disease, thiamine had a significant effect only in the quartile model (HR 0.29; 95% CI 0.09–0.91 highest vs. lowest; P trend 0.024) while vitamin B6 intake was associated with lowered risk only in the continuous model (HR 0.53; 95% CI 0.34–0.82). The test for heterogeneity between ER+PR+ and ER-PR- for vitamin B6 was significant (P = 0.040).

**Table 5** shows associations between vitamin intake and risk of HER2+/- breast cancer. The highest quartile of riboflavin intake, compared to lowest, was associated with significantly reduced risk of both HER2+ (HR 0.28; 95% CI 0.10–0.74; P trend 0.004) and HER2- (HR 0.47; 95% CI 0.29–0.76; P trend 0.002) disease.

**Table 5:** HRs and 95% CIs of breast cancer by HER2 status in relation to quartiles of B vitamin intake in ORDET women.

	HER2+		HER2-	
	Cases/person-year	Multivariable HR (95% CI)*	Cases/person-year	Multivariable HR (95% CI)
**Thiamine**				
I	17/33560	1	90/34183	1
II	20/33817	0.87 (0.44–1.75)	60/34132	0.66 (0.46–0.94)
III	11/33632	0.38 (0.55–0.92)	61/34013	0.67 (0.44–1.01)
IV	14/34055	0.31 (0.10–0.99)	62/34404	0.66 (0.38–1.15)
P trend**		0.017		0.120
Continuous #		0.88 (0.51–1.52)		0.80 (0.61–1.04)
P for Heterogeneity##	0.720			
**Riboflavin**				
I	23/33756	1	97/34356	1
II	14/33664	0.44 (0.22–0.89)	60/34054	0.58 (0.41–0.82)
III	10/33792	0.26 (0.11–0.61)	61/34151	0.57 (0.39–0.83)
IV	15/33852	0.28 (0.10–0.74)	55/24171	0.47 (0.29–0.76)
P trend		0.004		0.002
Continuous		0.67 (0.43–1.04)		0.82 (0.67–1.00)
P for Heterogeneity##	0.430			
**Niacin**				
I	15/33401	1	76/33911	1
II	14/33792	0.85 (0.40–1.82)	79/34334	1.04 (0.75–1.46)
III	17/33842	1.00 (0.45–2.22)	59/34153	0.80 (0.54–1.19)
IV	16/34029	0.89 (0.33–2.36)	59/34335	0.82 (0.51–1.32)
P trend		0.923		0.252
Continuous		0.91 (0.61–1.36)		0.82 (0.67–1.00)
P for Heterogeneity##	0.638			
**Vitamin B6**				
I	16/33542	1	87/34113	1
II	18/33653	0.96 (0.47–1.95)	72/34120	0.79 (0.56–1.09)
III	11/33822	0.55 (0.23–1.30)	56/34163	0.59 (0.40–0.87)
IV	17/34047	0.73 (0.27–1.96)	58/34337	0.58 (0.36–0.94)
P trend		0.324		0.011
Continuous		0.74 (0.48–1.14)		0.77 (0.63–0.95)
P for Heterogeneity##	0.862			
**Folate**				
I	16/33530	1	81/34106	1
II	16/33766	0.89 (0.43–1.85)	74/34238	0.89 (0.64–1.25)
III	15/33954	0.80 (0.36–1.76)	67/34306	0.79 (0.55–1.14)
IV	15/33813	0.78 (0.31–1.98)	51/34083	0.60 (0.38–0.94)
P trend		0.563		0.028
Continuous		0.92 (0.64–1.33)		0.87 (0.73–1.04)
P for Heterogeneity##	0.772			

* Adjusted for height, waist-hip-ratio, age at menarche, menopausal status, oral contraceptive use, parity, education, family history of breast cancer, energy intake, and alcohol intake.

** Tests for linear trend were calculated by assigning an ordinal number to each quartile.

# HR of developing breast cancer per 1 SD increase in vitamin intake.

## HER2+ vs. HER2-.

The highest quartile of thiamine intake, compared to lowest, was associated with significantly reduced risk of HER2+ disease (HR 0.31; 95% CI 0.10–0.99; P trend, 0.017).

Compared to the lowest, the highest quartiles of vitamin B6 (HR 0.58; 95% CI 0.36–0.94; P trend 0.011) and folate (HR 0.60; 95% CI 0.38–0.94 P trend 0.028) were associated with significantly lowered risk of HER2- disease. Vitamin B6 intake was also significantly associated with reduced risk of HER2- disease in the continuous model (HR 0.77; 95% CI 0.63–0.95). No significant associations of niacin, vitamin B6 or folate with HER2+ disease were found. No significant associations of niacin or thiamine with HER2- disease were found. Tests for heterogeneity between HER2+ and HER2- cancers were not significant for any B vitamin.

We further investigated the association of vitamin B with the risk of triple-negative cancers (ER-/PR-/HER2-, only 37 cases), but found no significant association between dietary intake of vitamin B and the risk of this poor prognosis breast cancer subtype (HR of 0.51; 95% CI 0.24–1.07 for thiamine, HR of 1.21; 95% CI 0.75–1.94 for riboflavin, HR of 0.69; 95% CI 0.40–1.21 for niacin, HR of 0.59; 95% CI 0.33–1.04 for vitamin B6 and HR of 1.05; 95% CI 0.67–1.65 for folate intake).

When the analyses were stratified by BMI, vitamin B6 was significantly associated with reduced risk in lean women whereas thiamine was associated with reduced risk in overweight women. Tests for heterogeneity were not significant (P = 0.088 for thiamine and P = 0.896 for vitamin B6) (S1 Table). In no case were associations between vitamin intake and breast cancer risk influenced by menopausal status (tests for interaction not significant). However, high vitamin B6 was significantly associated with reduced risk in premenopausal women, whereas high riboflavin was significantly associated with reduced risk both in pre- and post-menopausal women (S2 Table). Folic acid intake was associated with reduced breast cancer risk in women who did not consume, or consumed little (<12g/day) alcohol; however it is unlikely that our study had the power to adequately examine such associations (S3 Table).

## Discussion

In the present prospective study we found that high intakes of thiamine and B vitamins involved in one-carbon metabolism (folate, riboflavin, vitamin B6) were associated with significantly lowered breast cancer risk. Furthermore, high folate intake was associated with reduced risk of ER+, PR+, ER+PR+ and HER2- subtypes; high thiamine was associated with reduced risk of ER-PR- and HER2+ disease; high riboflavin was associated with reduced risk of ER+, PR+, HER2+, and HER2- disease; and high vitamin B6 was associated with reduced risk of ER+, ER-, PR-, and HER2- cancers.

We examine first folate. The literature on folate intake and breast cancer is extensive. Recent reviews/meta-analyses do not provide a clear picture [6,7]. Most cohort studies did not find an association of folate intake with breast cancer risk [8,9,12,14,28–34], but several case-control studies reported an apparent protective effect,[35], in agreement with the findings of the present study.

The literature (cohort studies) on folate intake and risk of breast cancer subtypes defined by ER and PR status is less extensive[8,12,13,30,34,36,37]; some reported null results [8,36], whereas the Nurses’ Health Study[37], the Vitamins And Lifestyle study [12], and the EPIC study (in premenopausal women only) [34] found that high folate intake was associated with lowered risk of ER- breast cancer. To our knowledge four cohort studies have assessed folate in relation to risk of breast cancer according to joint ER/PR status [30,34,38,39]. In the Swedish Mammography Cohort [30] folate intake was associated with a significantly reduced risk of ER+PR- breast cancer, but not of ER+PR+ or ER-PR- cancer. The Shanghai Women’s Health Study [38] and the EPIC study [34] did not find significant associations of folate with ER+PR+ or ER-PR- breast cancer, but Roswall et al.[39] reported that high dietary folate was associated with increased risk of ER+PR- breast cancer. We found that high folate intake was associated with reduced risk of ER+, PR+, and ER+PR+ cancers, and also that high folate tended to be associated with lowered risk of ER- breast cancer, but not significantly.

To our knowledge, only the EPIC study [34] has evaluated the relation of folate intake to the HER2 status of breast cancer, reporting null results. We found that high folate intake was associated with significantly lowered risk of HER2- breast cancer.

Folate plays an important role in DNA methylation [40,41] and is involved in the conversion of homocysteine to methionine and the production of S-adenosyl methionine (SAM)–an essential component of protein, RNA and DNA methylation reactions [42]. Low folate status may alter DNA methylation to influence gene expression, and adversely affect DNA integrity and stability [41]. DNA methylation occurs mainly on the cytosine of CpG islands and typically results in epigenetic downregulation of gene expression.

Methylation of CpG islands on the ER gene has been associated with lack of ER gene expression in breast cancer cell lines and primary breast cancers [43–46]. Hypermethylation has also been found to be involved in silencing PR gene expression [47]. However, findings from our study do not support the hypothesis that low folate intake is associated with increased risk of ER- breast cancer.

As regards riboflavin and vitamin B6, these are involved in one-carbon metabolism as enzyme cofactors [48]. Riboflavin is cofactor for the enzymes methylenetetrahydrofolate reductase (MTHFR) and methionine synthase reductase (MTRR), while vitamin B6 is cofactor for cystathionine β-synthase and cystathionine γ-lyase [49]. Results from cohort studies that investigated the relation of these vitamins to breast cancer risk have produced inconsistent results [8,12,13,32,34,50,51]. To our knowledge three cohort studies have examined relations between the ER, PR and combined ER-PR status of breast cancers and riboflavin [12,38] and vitamin B6[8,12,38] reporting no association. We found that high riboflavin intake was associated with significantly reduced risk of ER+PR+, HER2+ and HER2- breast cancers, so that the apparent protective effect of riboflavin was independent of HER2 status. We also found that high vitamin B6 was associated with reduced risk of HER2- breast cancer.

Vitamin B6 and riboflavin may protect against breast cancer by mechanisms unrelated to one-carbon metabolism. It is known that chronic inflammation plays a role in the development and progression of several types of cancer [52] including breast cancer [53]. Vitamin B6 is required for the production of inflammation-mediating cytokines [54] and the activation of lymphocytes as part of the inflammatory response [55]. So it is of interest that several inflammatory conditions have been associated with low plasma levels vitamin B6, although the significance of this association remains unclear [56].

As regards thiamine, this enzyme cofactor is involved in metabolic processes that are often altered in tumour tissue [57,58]. However the few studies that investigated thiamine intake in relation to breast cancer risk found no significant association [9,32,59]; and no studies appear to have investigated thiamine intake and risk of specific breast cancer subtypes. In the present study we found that high thiamine intake was associated with significantly lowered risks of developing breast cancer in general, and also of PR-, ER-PR- and HER2+ subtypes. However, further studies are required to confirm these associations and elucidate their mechanisms.

The strengths of the present study are its prospective design, and highly complete follow-up. Furthermore, dietary habits, over the year prior to assessment, were assessed using a food frequency questionnaire that was designed to capture the eating habits of the enrolled women by including local recipes and products. The main sources of folate in our cohort were vegetables (28.0%), cereals (17.4%) and fruits (12.2%); for riboflavin they were milk and dairy products (24.5.0%), meat and meat products (21.8%), and vegetables (15.2%); for vitamin B6 they were meat and meat products (28.9%), cereals (16.7%) and fruits (15.3%); for thiamine they were cereals (23.0%), meat and meat products (22.0%), and fruits (14.0%). Given the wide distribution of sources for these micronutrients it is unlikely that the risk reductions can be attributed to a specific dietary pattern (for example a Mediterranean-type diet) or to generic factors such as overall healthy lifestyle, however we cannot exclude these possibilities.

A limitation is that only a single dietary assessment was made and any changes in dietary habits since that assessment (20 years ago) will have been missed. Furthermore, we cannot rule out confounding by factors that we were not able to estimate or estimated in a sub-optimal way in our questionnaires.

Another limitation is the hormone receptor expression of the cancers was determined using various biochemical and immunohistochemical techniques that may not be completely equivalent to each other, resulting in some misclassification receptor status.

To conclude, our study supports the hypothesis that high dietary intakes of folate and other B vitamins involved in one-carbon metabolism reduce breast cancer risk. It also suggests that folate protects against ER+PR+ and HER2- subtypes; and that thiamine protects against be ER-PR- and HER2+ subtypes. However further prospective studies are required to clarify the associations of these vitamins with risk of developing different breast cancer subtypes.

## Supporting Information

S1 Table HRs (95% CIs) of breast cancer in relation to quartiles of B vitamin intake in ORDET women, stratified by BMI (DOCX)

S2 Table HRs (95% CIs) of breast cancer in relation to quartiles of B vitamin intake in ORDET women, stratified by menopausal status (DOCX)

S3 Table HRs (95% CIs) of breast cancer in relation to quartiles of folate intake in ORDET women, stratified by alcohol consumption (DOCX)
