# Supplementary material for: Micronutrients Involved in One-Carbon Metabolism and Risk of Breast Cancer Subtypes
Source: PLoS One. 2015 Sep 16;10(9):e0138318. doi: 10.1371/journal.pone.0138318 (PMC4574438; doi:10.1371/journal.pone.0138318)
Supplement: S1 Table — (DOCX) [file pone.0138318.s001.docx]

| **Table S1. HRs (95% CIs) of breast cancer in relation to quartiles of B vitamin intake in ORDET women. stratified by BMI** | | | | |
| --- | --- | --- | --- | --- |
|  | **BMI≤25** | | **BMI>25** | |
|  | **Cases/**  **Non-cases** | **Multivariate**  **RR (95% CI)*** | **Cases/**  **Non-cases** | **Multivariate**  **RR (95% CI)*** |
| **Thiamine** |  |  |  |  |
| I | 52/1099 | 1 | 70/1057 | 1 |
| II | 59/1184 | 112 (0.75 – 1.69) | 37/973 | 0.47 (0.31 - 0.73) |
| III | 47/1228 | 0.93 (0.56 – 1.52) | 39/921 | 0.46 (0.28 - 0.76) |
| IV | 51/1134 | 1.20 (0.63 - 2.27) | 35/1011 | 0.28 (0.14 - 0.56) |
| P for trend^**^ |  | 0.844 |  | 0.000 |
| Continuous ^***^ | 209/4645 | 1.07 (0.78 - 1.45) | 181/3962 | 0.69 (0.49 - 0.96) |
| P for Heterogeneity |  | 0.088 |  |  |
| **Riboflavin** |  |  |  |  |
| I | 76/1143 | 1 | 64/1013 | 1 |
| II | 47/1244 | 0.52 (0.35 - 0.77) | 39/910 | 0.63 (0.41 - 0.96) |
| III | 41/1148 | 0.47 (0.30 - 0.73) | 39/1004 | 0.53 (0.33 - 0.83) |
| IV | 45/1110 | 0.48 (0.28 - 0.81) | 39/1035 | 0.47 (0.26 - 0.83) |
| P for trend** |  | 0.004 |  | 0.005 |
| Continuous *** | 209/4645 | 0.85 (0.67 - 1.08) | 181/3962 | 0.73 (0.57 - 0.93) |
| P for heterogeneity |  | 0.901 |  |  |
| **Niacin** |  |  |  |  |
| I | 55/1165 | 1 | 50/988 | 1 |
| II | 59/1211 | 1.04 (0.71 - 1.54) | 47/942 | 0.98 (0.64 - 1.48) |
| III | 48/1171 | 0.91 (0.58 - 1.43) | 43/981 | 0.90 (0.56 - 1.45) |
| IV | 47/1098 | 1.00 (0.58 - 1.72) | 41/1051 | 0.82 (0.46 - 1.48) |
| P for trend** |  | 0.838 |  | 0.496 |
| Continuous *** | 209/4645 | 0.89 (0.70 - 1.12) | 181/3962 | 0.87 (0.68 - 1.11) |
| P for heterogeneity |  | 0.724 |  |  |
| **Vitamin B6** |  |  |  |  |
| I | 61/1159 | 1 | 58/995 | 1 |
| II | 60/1220 | 0.87 (0.59 - 1.26) | 40/932 | 0.72 (0.47 - 1.09) |
| III | 49/1176 | 0.69 80.44 - 1.08) | 40/977 | 0.67 (0.42 - 1.07) |
| IV | 39/1090 | 0.56 (0.31 - 0.99) | 43/1058 | 0.66 (0.36 - 1.19) |
| P for trend** |  | 0.034 |  | 0.134 |
| Continuous *** | 209/4645 | 0.76 (0.60 - 0.97) | 181/3962 | 0.79 (0.61 - 1.01) |
| P for heterogeneity |  | 0.846 |  |  |
| **Folate** |  |  |  |  |
| I | 59/1173 | 1 | 53/980 | 1 |
| II | 63/1212 | 1.00 (0.69 - 1.45) | 44/941 | 0.85 (0.56 - 1.30) |
| III | 47/1165 | 0.77 (0.50 - 1.19) | 48/987 | 0.84 (0.54 - 1.31) |
| IV | 40/1095 | 0.70 (0.41 - 1.18) | 36/1054 | 0.59 (0.34 - 1.03) |
| P for trend** |  | 0.114 |  | 0.092 |
| Continuous *** | 209/4645 | 0.95 (0.78 - 1.17) | 181/3962 | 0.80 (0.64 - 0.99) |
| P for heterogeneity |  | 0.878 |  |  |
| * Adjusted for height. waist-hip-ratio. age at menarche. menopausal status. oral contraceptive use. parity. education. family history of breast cancer. energy intake. and alcohol intake.  ** Tests for linear trend calculated by assigning an ordinal number to each quartile.  *** HR of developing breast cancer per 1 SD increase in vitamin intake | | | | |
