# Supplementary material for: Micronutrients Involved in One-Carbon Metabolism and Risk of Breast Cancer Subtypes
Source: PLoS One. 2015 Sep 16;10(9):e0138318. doi: 10.1371/journal.pone.0138318 (PMC4574438; doi:10.1371/journal.pone.0138318)
Supplement: S2 Table — (DOCX) [file pone.0138318.s002.docx]

| **Table S2. HRs (95% CIs) of breast cancer in relation to quartiles of B vitamin intake in ORDET women, stratified by menopausal status** | | | | |
| --- | --- | --- | --- | --- |
|  | **Pre-menopause** | | **Post-menopause** | |
|  | **Cases/**  **Non-cases** | **Multivariate**  **RR (95% CI)*** | **Cases/**  **Non-cases** | **Multivariate**  **RR (95% CI)*** |
| **Thiamine** |  |  |  |  |
| I | 70/1389 | 1 | 69/1287 | 1 |
| II | 69/1626 | 0.84 (0.58 - 1.22) | 46/1063 | 0.80 (0.52 - 1.23) |
| III | 71/1841 | 0.75 (0.45 - 1.25) | 40/821 | 0.90 (0.47 - 1.70) |
| P for trend** |  | 0.262 |  | 0.617 |
| Continuous *** | 210/4856 | 0.81 (0.60 - 1.10) | 155/3171 | 0.97 (0.67 - 1.40) |
| P for Heterogeneity |  | 0.640 |  |  |
| **Riboflavin** |  |  |  |  |
| I | 86/1425 | 1 | 75/1257 | 1 |
| II | 54/1661 | **0.52 (0.36 - 0.75)** | 43/1010 | **0.63(0.42 - 0.95)** |
| III | 70/1770 | **0.59 (0.38 - 0.92)** | 37/904 | **0.54 (0.32 - 0.92)** |
| P for trend** |  | **0.013** |  | **0.016** |
| Continuous *** | 210/4856 | 0.81 (0.65 - 1.02) | 155/3171 | **0.75 (0.57 - 0.98)** |
| P for Heterogeneity |  | 0.912 |  |  |
| **Niacin** |  |  |  |  |
| I | 73/1472 | 1 | 71/1206 | 1 |
| II | 72/1590 | 0.91 (0.64 - 1.29) | 41/1084 | **0.62 (0.40 - 0.94)** |
| III | 65/1794 | 0.71 (0.46 - 1.12) | 43/881 | 0.73 (0.43 - 1.25) |
| P for trend** |  | 0.149 |  | 0.169 |
| Continuous *** | 210/4856 | 0.85 (0.68 - 1.06) | 155/3171 | 0.95 (0.72 - 1.24) |
| P for Heterogeneity |  | 0.366 |  |  |
| **Vitamin B6** |  |  |  |  |
| I | 77/1469 | 1 | 68/1214 | 1 |
| II | 75/1608 | 0.80 (0.56 - 1.12) | 42/1068 | 0.68 (0.45 - 1.05) |
| III | 58/1779 | **0.50 (0.31 - 0.80)** | 45/889 | 0.88 (0.51 - 1.53) |
| P for trend** |  | **0.005** |  | 0.511 |
| Continuous *** | 210/4856 | **0.71 (0.57 - 0.90)** | 155/3171 | 0.85 (0.64 - 1.12) |
| P for Heterogeneity |  | 0.217 |  |  |
| **Folate** |  |  |  |  |
| I | 70/1496 | 1 | 68/1180 | 1 |
| II | 83/1636 | 1.07 (0.76 - 1.51) | 47/1040 | 0.73 (0.49 - 1.09) |
| III | 57/1724 | 0.71 (0.45 - 1.11) | 40/951 | 0.62 (0.38 - 1.03) |
| P for trend** |  | 0.147 |  | 0.058 |
| Continuous *** | 210/4856 | 0.86 (0.70 - 1.05) | 155/3171 | 0.82 (0.65 - 1.04) |
| P for Heterogeneity |  | 0.946 |  |  |
| * Adjusted for height. waist-hip-ratio. age at menarche. menopausal status. oral contraceptive use. parity. education. family history of breast cancer. energy intake. and alcohol intake.  ** Tests for linear trend calculated by assigning an ordinal number to each quartile.  *** HR of developing breast cancer per 1 SD increase in vitamin intake | | | | |
