# Supplementary material for: Micronutrients Involved in One-Carbon Metabolism and Risk of Breast Cancer Subtypes
Source: PLoS One. 2015 Sep 16;10(9):e0138318. doi: 10.1371/journal.pone.0138318 (PMC4574438; doi:10.1371/journal.pone.0138318)
Supplement: S3 Table — (DOCX) [file pone.0138318.s003.docx]

| **Table S3. HRs (95% CIs) of breast cancer in relation to quartiles of folate intake in ORDET women, stratified by alcohol consumption** | | | | | | |
| --- | --- | --- | --- | --- | --- | --- |
|  | **Alcohol consumption** | | | | | |
|  | **Abstainer** | | **Medium consumption**  **(≤12g/day)** | | **High consumption**  **(>12g/day)** | |
|  | **Cases/**  **Non-cases** | **Multivariate**  **RR (95% CI)*** | **Cases/**  **Non-cases** | **Multivariate**  **RR (95% CI)*** | **Cases/**  **Non-cases** | **Multivariate**  **RR (95% CI)*** |
| **All BC** |  |  |  |  |  |  |
| **Folate tertiles** |  |  |  |  |  |  |
| I | 64/1220 | 1 | 31/580 | 1 | 51/1073 | 1 |
| II | 66/1182 | 0.96 (0.66 - 1.40) | 26/609 | 0.68 (0.39 - 1.19) | 49/1082 | 1.11 (0.73 - 1.70) |
| III | 37/1163 | **0.53 (0.32 - 0.88)** | 23/635 | **0.48 (0.23 - 0.97)** | 44/1074 | 1.11 (0.66 - 1.87) |
| P for trend** |  | **0.020** |  | **0.039** |  | 0.675 |
| Continuous*** | 167/3565 | **0.77 (0.62 - 0.97)** | 80/1824 | **0.68 (0.48 - 0.95)** | 144/3229 | 1.12 (0.89 - 1.42) |
|  |  |  |  |  |  |  |
| * Adjusted for height, waist-hip-ratio, age at menarche, menopausal status, oral contraceptive use, parity, education, family history of breast cancer, energy intake, and alcohol intake.  ** Tests for linear trend calculated by assigning an ordinal number to each quartile.  *** HR of developing breast cancer per 1 SD increase in vitamin intake | | | | | | |
